# Spatio-Temporal Dynamics of Exploited Groundfish Species Assemblages Faced to Environmental and Fishing Forcings: Insights from the Mauritanian Exclusive Economic Zone

**DOI:** 10.1371/journal.pone.0141566

**Published:** 2015-10-27

**Authors:** Saïkou Oumar Kidé, Claude Manté, Laurent Dubroca, Hervé Demarcq, Bastien Mérigot

**Affiliations:** 1 Institut Mauritanien de Recherches Océanographiques et des Pêches, Laboratoire de Biologie et Ecologie des Organismes Aquatiques, Nouadhibou, Mauritanie; 2 Aix-Marseille Université, Mediterranean Institute of Oceanography, Marseille, France; 3 IFREMER, Avenue du Général de Gaulle, 14520, Port-en-Bessin-Huppain, France; 4 Institut de Recherche pour le Développement (IRD), UMR 9190 MARBEC, Centre de Recherche Halieutique Méditerranéenne, Sète, France; 5 Université de Montpellier, UMR 9190 MARBEC, Centre de Recherche Halieutique Méditerranéenne, Sète, France; Hellenic Centre for Marine Research, GREECE

## Abstract

Environmental changes and human activities can have strong impacts on biodiversity and ecosystem functioning. This study investigates how, from a quantitative point of view, simultaneously both environmental and anthropogenic factors affect species composition and abundance of exploited groundfish assemblages (*i*.*e*. target and non-target species) at large spatio-temporal scales. We aim to investigate (1) the spatial and annual stability of groundfish assemblages, (2) relationships between these assemblages and structuring factors in order to better explain the dynamic of the assemblages’ structure. The Mauritanian Exclusive Economic Zone (MEEZ) is of particular interest as it embeds a productive ecosystem due to upwelling, producing abundant and diverse resources which constitute an attractive socio-economic development. We applied the multi-variate and multi-table STATICO method on a data set consisting of 854 hauls collected during 14-years (1997–2010) from scientific trawl surveys (species abundance), logbooks of industrial fishery (fishing effort), sea surface temperature and chlorophyll a concentration as environmental variables. Our results showed that abiotic factors drove four main persistent fish assemblages. Overall, chlorophyll a concentration and sea surface temperature mainly influenced the structure of assemblages of coastal soft bottoms and those of the offshore near rocky bottoms where upwellings held. While highest levels of fishing effort were located in the northern permanent upwelling zone, effects of this variable on species composition and abundances of assemblages were relatively low, even if not negligible in some years and areas. The temporal trajectories between environmental and fishing conditions and assemblages did not match for all the entire time series analyzed in the MEEZ, but interestingly for some specific years and areas. The quantitative approach used in this work may provide to stakeholders, scientists and fishers a useful assessment for the spatio-temporal dynamics of exploited assemblages under stable or changing conditions in fishing and environment.

## Introduction

Faced with natural changes and human activities, marine resources management need to adopt an integrated view of ecosystems. Since the productivity of marine resources by fisheries depends on the ecological state of ecosystems (not only the dynamics of target species, but also the dynamics of non-target organisms) environmental factors and human impacts have to be considered [1,2]. This can be achieved in the framework of the Ecosystem-based Approach to Fisheries (EAF) [3,4,5].

Some ecological studies brought important contributions in recent decades around the world on the processes that structure target species [6,7,8], non-target species [2,9,10], and marine exploited fish assemblages as a whole [11,12]. Recent studies have allowed improvements in the understanding of the changes of groundfish assemblages in response to various factors such as fishing or environmental changes in marine areas [13,14,15,16]. These forcings have broad and varied impacts on the fish species, including the variability in abundance, productivity and the composition of assemblages [17,18,19,20]. However, during the past two decades, studies around the world have been focused separately on the effects of fishing on the structure of exploited assemblages [21,22,23,24], while others were dedicated to the effects of environmental variables [25,26]. To our knowledge, there is a lack of studies that investigate simultaneously from a quantitative point of view the effects of both environmental and anthropogenic factors with a focus on the species composition and abundance of exploited groundfish assemblages at large spatio-temporal scales.

In this context, the Mauritanian Exclusive Economic Zone (MEEZ) is a particularly interesting case study for its environmental and demersal fisheries characteristics. It is strongly affected by hydrographic features, notably under the influence of two ocean currents. These currents and the profile of the continental shelf trigger an important upwelling phenomenon. This oceanographic phenomenon lasts 12 months in the area of Cap Blanc [27,28] and in the South of Cap Blanc, while it is seasonal from December to March in the area adjacent to Nouakchott [29,30,31]. It provides an area of high plankton productivity and supports a large variety of fish communities with many commercial species that sustain various fishing activities [32,33].

Despite these important environmental features, the first attempts on the characterization of fish assemblages are limited to a part of the coast and remain spatially fragmented [34]. Indeed, these studies were restricted to the north part of Western Sahara and of the southern part of Mauritania [35], near the Cap Blanc [36,37], or even the Banc d’Arguin area [38,39,40]. Available works over the coast were performed by Jouffre and Inejih [41]. Several studies were done in deeper waters in the North Western African region on the continental shelf and the slope [42,43,44,45].

This study is a contribution to investigate how, from a quantitative point of view, simultaneously both environmental and anthropogenic factors affect species composition and abundance of exploited groundfish assemblages’ (both target and non-target species) at large spatial and temporal scales, with the case study of the MEEZ. More precisely, we aim at investigating (1) the spatial and annual stability of assemblages of groundfish, and (2) relationships between these assemblages and structuring factors (fishing effort, chlorophyll a concentration, temperature) in order to better explain the dynamic of the assemblages’ structure. Our work is based on statistical analyses performed on a huge data set consisting of 854 hauls collected within different depth strata and latitudinal areas during 14-years (1997–2010) of scientific trawl surveys (species abundance data), logbooks of industrial fishery (fishing effort), satellite data (sea surface temperature and chlorophyll a) as environmental variables.

## Materials and Methods

The trawling surveys to enhance demersal ressources estimates within several research studies were among the research priorities of the Mauritanian Institute of Oceanographic Research and Fisheries (IMROP) under the approval of the Ministry of Fisheries and Maritime Economy (MPEM, Law number 2000–025 Code of Fisheries, Chapter 3, Article 30). After being reported in the database, if protected species were caught during samplings a particular attention was paid to release them alive when possible.

### Study area

**Fig 1:**
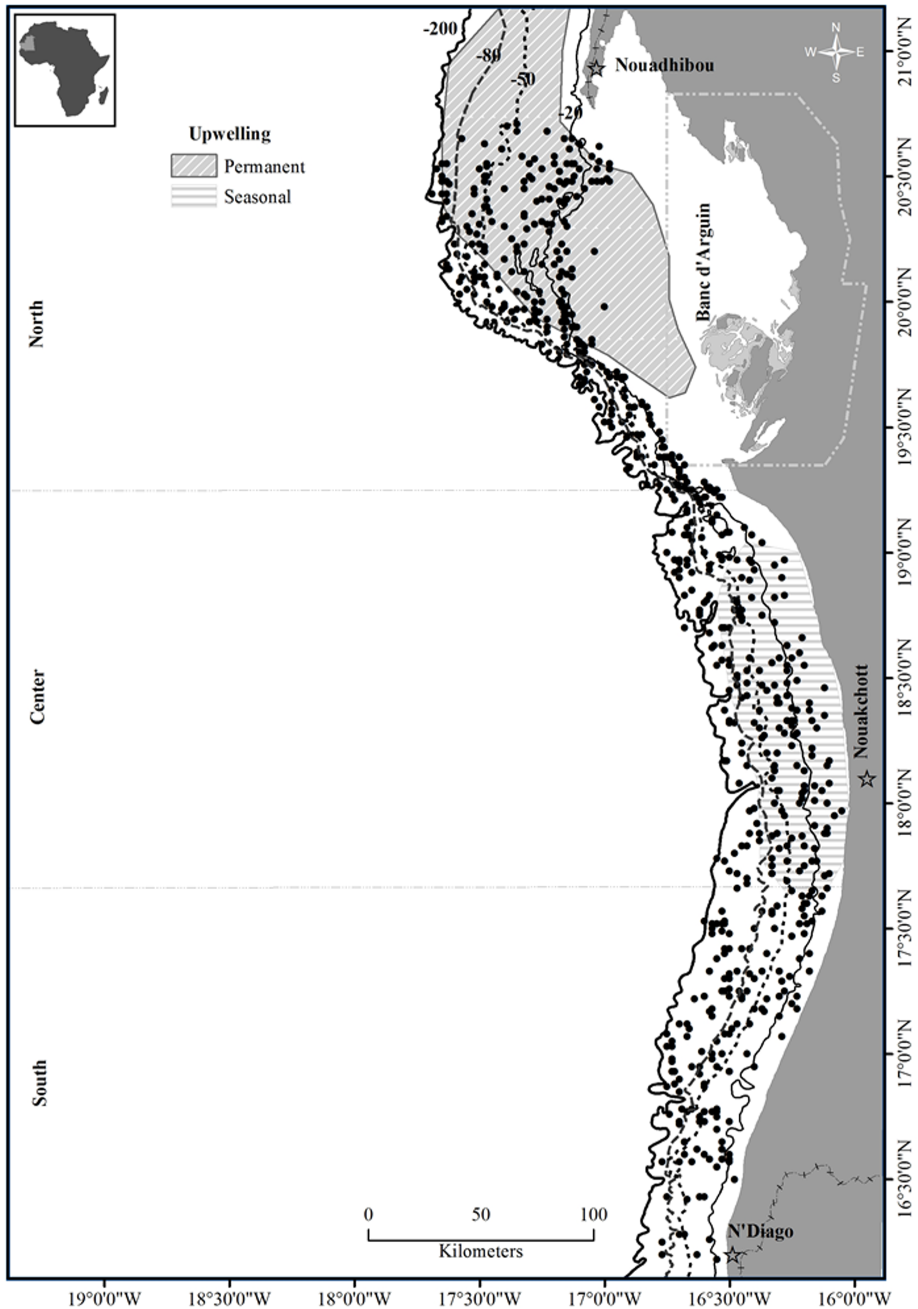
Map of trawl stations. Their locations (black points) are within four different bathymetric strata between 0 to 200m depth (isobaths are in dotted lines).

The Mauritanian coast is situated on the Atlantic side of the northwestern African continent. The continental shelf covers a distance of approximately 750 km and an area of 36 000 km2 with an exclusive economic zone of 230 000 km2. The study area extends from 16°05’N in the South with the border of Senegal and up to 20°36’N in the North at the Western Sahara area. The trawling stations are located in three main areas (North, Center and South, Fig 1, see below for their contrasted environmental and fishing conditions), and they were divided into four main bathymetric strata: namely (1) coastal shelf (CS: 0–20 m); (2) upper shelf (US: 20–50 m); (3) mid-shelf (MS: 50–80 m); (4) and outer shelf (OS: 80–200 m deep) (Fig 1). These bathymetric strata reflect trends in substrate characteristics and seasonal variations in hydrological conditions [46,47,48].

### Data collected

In our study the scientific trawl stations represent the statistical units (individuals in data tables). Biological variables (*i*.*e*. count of fish species) have been taken from abundance data collected during seasonal scientific trawl surveys performed on the continental shelf (<200 m depth). Influence of environmental variables (sea surface temperature and chlorophyll a concentration) and exploitation effort of the fishing industry (number of fishing operation by areas) are investigated.

### Fish abundances

Demersal fish abundances were obtained from scientific trawl surveys performed by oceanographic vessel (Al Awam) of IMROP in which participated S.O. Kidé. The sampling method consists of a random stratified sampling design [46]. The trawl used throughout surveys was a polyethylene bottom trawl net of “Irish” type with a 45 mm codend, and a 60 mm mesh in the wings. The gear has a horizontal opening of 17.5 m and a vertical opening varying between 2.8 to 3.5 m. Trawling speed varied between 2.5 and 3.95 knots, and the duration of fishing ranged from 15 to 40 minutes. Abundance data were standardized per half an hour of trawling in order to adjust variability in trawling duration. All the species captured in a given station were identified, counted and then recorded on the database. The sampling strategy and the observation protocol remained the same during the 14 years of the study.

Groundfish assemblages sampled in the MEEZ consisted of 543 fish species, belonging to 322 genera and 176 families on the continental shelf during the study period. We focused our study only on 71 groundfish species (Chondrichthyes and Osteichthyes) properly sampled by trawling, and which appeared at least 5% on the data set (for accuracy with the statistical method used below).

### Environmental and fishing effort data

Environmental variables, sea surface temperature (SST°C) and chlorophyll a concentration (Chl a mg/m^3^), were obtained from satellite data. SST used is from the version 5 of the AVHRR Oceans Pathfinder SST data set obtained from the Physical Oceanography Distributed Active Archive Center (PO.DAAC) at the NASA Jet Propulsion Laboratory, Pasadena, CA. http://podaac.jpl.nasa.gov. Eight-day averaged day-time SST at 4.5 km resolution was extracted for the period 1997–2010 in order to match the study area. The sea surface Chl a concentration were similarly extracted from the SeaWiFS 8-day-time archive for the period 1997 to 2010 at the 4.5 km resolution, from the 2009.1 reprocessing data set made available from NASA at http://oceandata.sci.gsfc.nasa.gov/. Both variables were extracted on the basis of the trawl positions. For each variable we used the median value of the 3x3 pixels area (about 14 km wide) centered in the trawl position for the nearest 8-day period of the trawling date.

The fishing logbook database describes catches and effort (ship characteristics, fishing position, type of license, duration of the tide, types of gear, number of operations, duration of fishing, species and quantities caught) of industrial fishing vessels (national and foreigners) with license of access to the resource in the MEEZ. The fishing effort was defined as the monthly average of the number of fishing operations (OpNu) in statistical squares of 0.5 by 0.5 degrees in latitude and longitude. This effort is used as a proxy of the anthropogenic pressure in this ecosystem.

**Fig 2:**
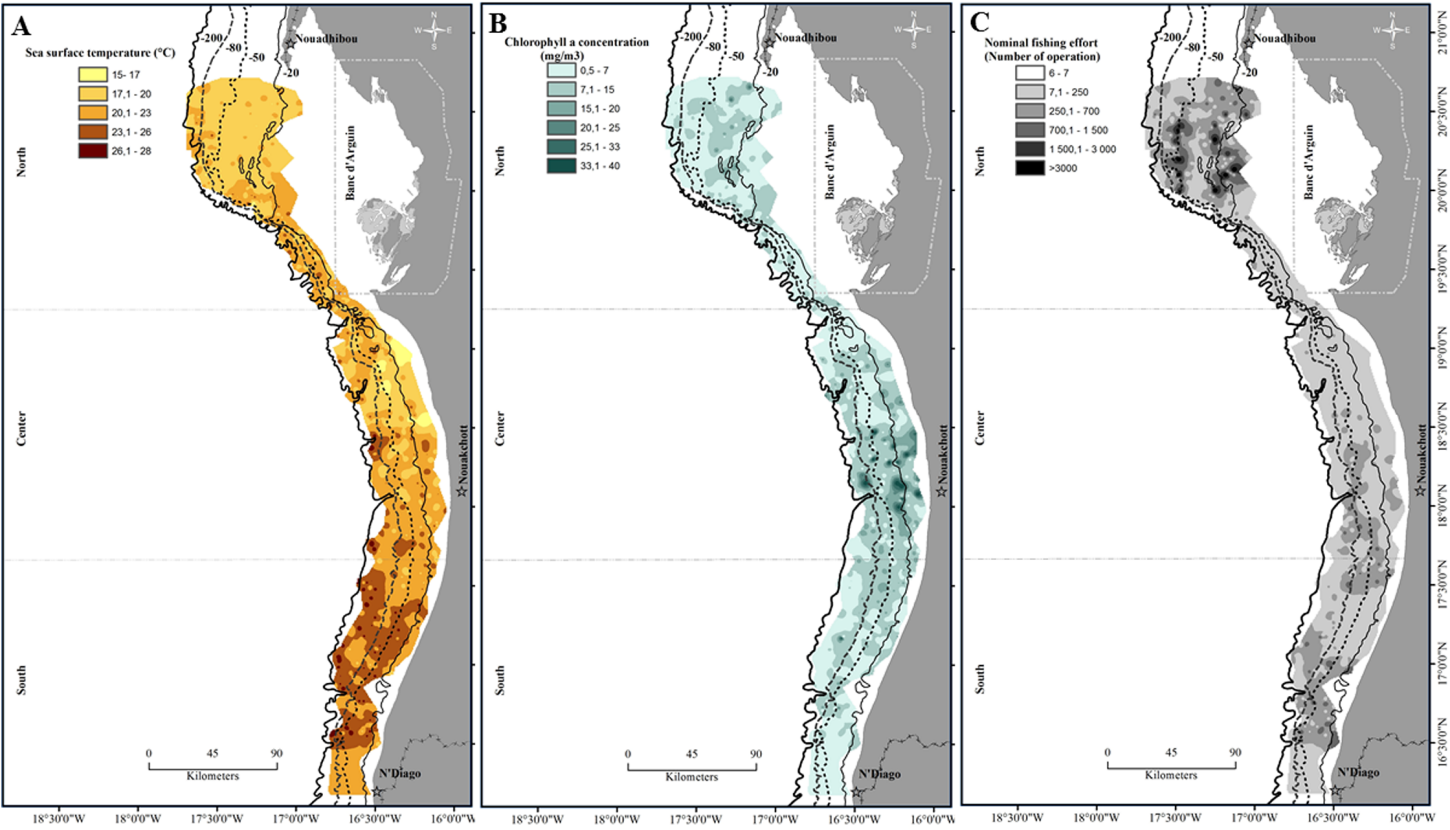
Mean annual spatial distribution of environmental and fishing variables on the Mauritanian continental shelf. (A) Sea surface temperature SST (°C), (B) chlorophyll a concentration Chl a (mg/m^3^) and (C) Fishing effort (Operation numbers OpNu) during 1997 to 2010.

Maps of the mean annual values between 1997 to 2010 of the SST, Chl a and fishing effort are provided in Fig 2. The southern zone is characterized by warm surface waters (annual average SST of 23.63 ± 2.68°C), Chl a (6.08 ± 5.74 mg/m3) and a variable fishing effort (347 ± 420 fishing operations). The central area encompassed lower SST (20.18 ± 2.67°C), Chl a relatively higher (7.13 ± 5.53 mg/m3) and a lower fishing effort (100 ± 91 operation numbers). In the northern area, SST was colder (19.16 ± 1.67°C), Chl a (7.14 ± 4.69 mg/m3) and higher fishing effort (429 operation numbers) were high.

### STATICO

The STATICO (STATIS and CO-inertia) method [49] was performed to describe the stable patterns and the spatio-temporal changes of the relationships between groundfishes and the environmental-fishing variables. To study the influence of bathymetry on the distribution of demersal fish and their dynamics over time, data were organized in a series of pairs of tables associated with the four depth strata, where individuals represent sampling year surveys. For each depth stratum, a table corresponded to yearly averages of the environmental (SST and Chl a) and fishing effort data (OpNu) while another table corresponded to the yearly sampled abundances of the 71 groundfish species variables.

The 4 pairs of tables correspond to the compiled bathymetric strata over a period of 14 years of sampling (1997 to 2010). Species abundances n were log (n+ 1) transformed to reduce the influence of too dominant species. Environment-fishing data were centered and reduced in order to consider their different units.

**Fig 3:**
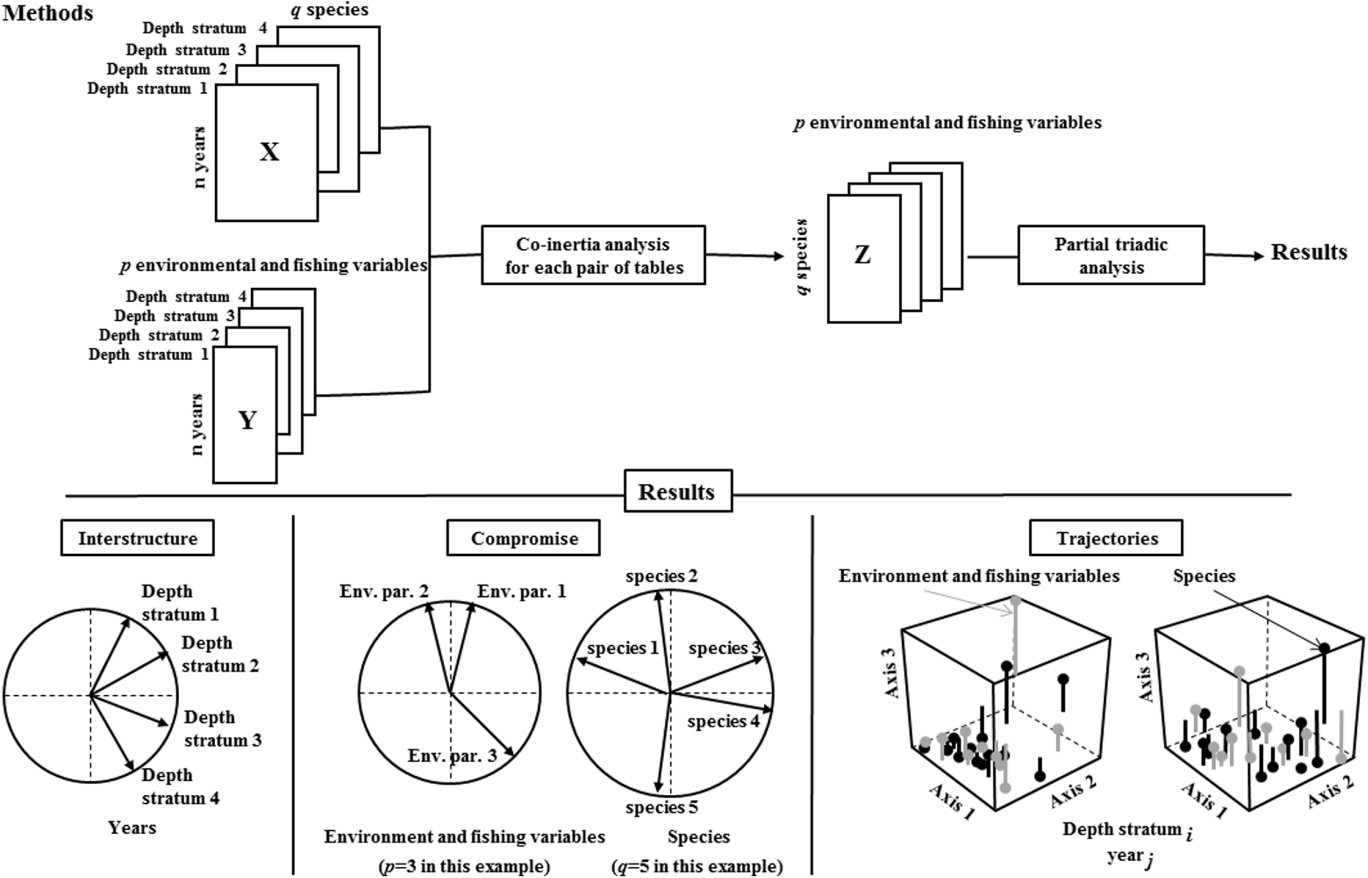
STATICO flow chart, the data structure is a sequence of n pairs of ecological tables. The X and Y are respectively the pairs of tables (species and environment-fishing). The Z is cross-table, *q* number of species, *p* number of variables environment-fishing. 1- Principal Component Analysis executed on each table (PCA) log transformed of species abundance and environment-fishing. 2- Co-inertia analyses allowing the link between the pairs of PCA, producing a sequence of cross-tables. 3- Partial Triadic Analysis (PTA) is used to analyze this series.

STATICO is an application of the STATIS (Structuration des Tableaux à Trois Indices de la Statistique; [50]) method called Partial Triadic Analysis (PTA; [51]) to co-inertia operators [52]. In other words, STATICO “combines the objectives of STATIS (finding the stable part of the structure of a series of tables) and the objectives of co-inertia analysis (finding the common structure of two data tables)”, as stated by Thioulouse et al. [49]. The aim of PTA is thus to identify the shared structure of a series of tables having same rows and same columns. A synthesis of the STATICO analysis is presented in the flow chart (Fig 3), and the description of the vectorial approach of the method is available in S1 Text. The data structure is a sequence of pairs of tables with the environmental-fishing variables and, separately, the species abundances. Each pair of tables is first linked by a co-inertia analysis [52,53]. Co-inertia analysis is a two-table coupling method, which allows a cross-table to be computed between the variables of the two tables (here between species and environmental variables). The resulting series of species and environmental variables cross-tables is then analyzed with a PTA leading to three main results/steps: (1) the interstructure step identifies the proximity between each pair of tables (in our case, the bathymetric strata); (2) the compromise analysis gives an ordination of the environmental variables and of the species on shared axes, and represents the average species-environment relationships across the years and shows the stable part of these relationships; (3) the trajectories step where species and environmental variables for each year can be projected as additional elements on the compromise axes in order to summarize the reproducibility of the structure across the series of cross-tables. For the sake of clarity, a clustering analysis was added at step (2) to identify groups of species on the compromise (see [54] for a similar approach). Indeed, on the factor map of the species, similar groups were identified with a hierarchical classification based on Euclidean matrix distance of pairwise distances between species on the three first factorial planes of the compromise analysis, using the UPGMA aggregation criterion (chosen with an objective approach among the main available aggregation criterion (see [55]). The optimal number of species group was identified with the Gap statistic [56].

Spatial distribution of trawl stations, environmental and fishing variables (Figs 1 and 2) were plotted using ArcGis 10 software (version 10.0, ESRI, Inc.). All statistical analyses were performed using the R environment [57]. R script is freely available as S1 File.

## Results

### Interstructure on the groundfish assemblages

**Fig 4:**
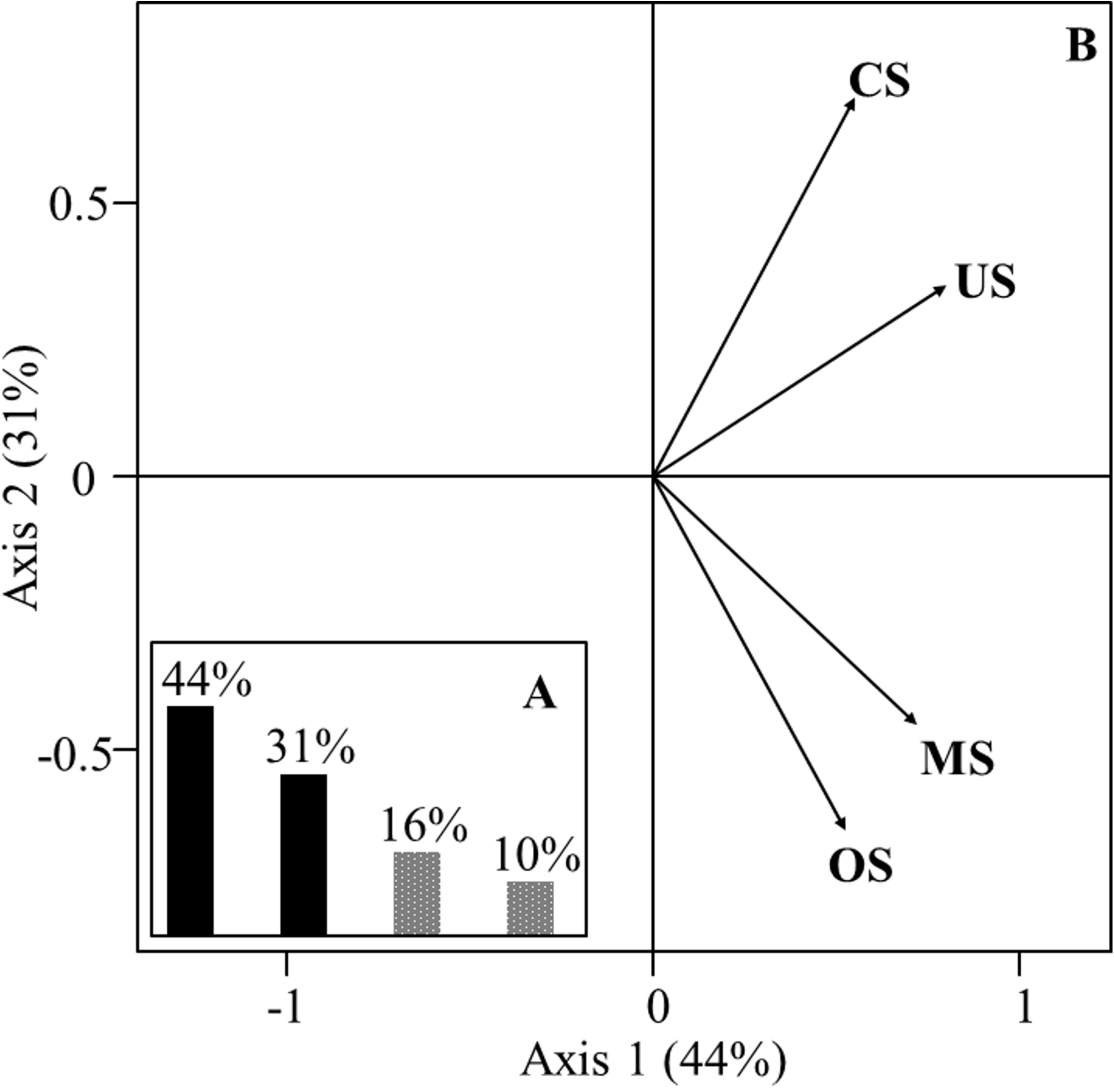
Interstructure plots to study spatial variability of environment-fishing variables and species assemblage on bathymetric strata. (A) Eigenvalues histogram of vector covariance matrix. (B) Projection tables (bathymetric strata) on the first factor plane of the compromise. Four tables pairs as bathymetric strata: coastal shelf (CS), upper shelf (US), mid-shelf (MS) and outer shelf (OS).

The interstructure is displayed on Fig 4. First the common structure associated with the compromise (first axis of the interstructure) explains 44% of the total variance (Fig 4A). The contributions (weights) of the depth strata to the compromise are positive and well-balanced (they range from 0.40 to 0.61 with a standard deviation of 0.09), and vector variances are similar to each other (see Table 1). Consequently the compromise is really sound. Along the second axis (31% of the total variance), two groups of strata depths are identified: coastal strata (CS and US) on the one hand and deeper strata (MS and OS) on the other hand (Fig 4B).

**Table 1:** Numerical variables associated with the STATICO analysis.

	RV = Correlation matrix						
Depth stratum	CS	US	MS	OS	*α*_*K*_	Cos2	Vect.Var.
CS	1				0.42	0.66	1.75
US	-0.01	1			0.40	0.42	1.22
MS	0.04	0.41	1		0.55	0.67	0.63
OS	0.48	0.13	0.38	1	0.61	0.82	0.40

Depth strata: coastal shelf (CS), upper shelf (US), mid-shelf (MS) and outer shelf (OS). RV coefficients of the vector covariance matrix between tables; α_K_: contribution of each table in the compromise and (cos2); cos2: fit of each table to the compromise; and Vect. Var.: Vector variance measuring the inertia of each table (depth stratum)

### Compromise on environment-fishing variables

**Fig 5:**
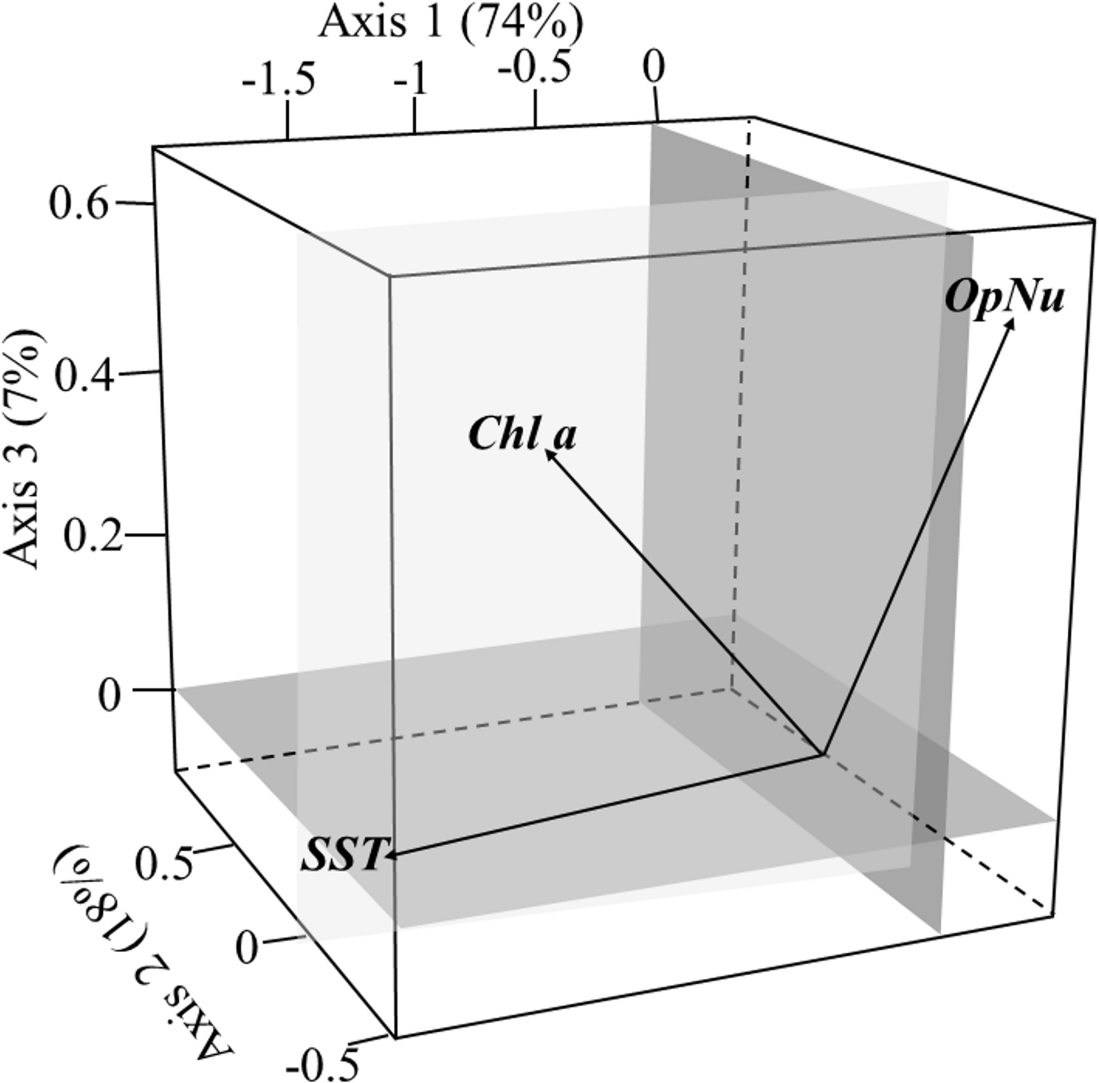
Compromise analysis on environment-fishing variables. (A) Sea surface temperature (*SST*). (B) chlorophyll a concentration (*Chl a*). (C) number of fishing operations (*OpNu*) onto the three first factor planes.

The first three axes of the analysis, representing 99% of the total inertia, were accounted to explain the variability of the common structure on environmental-fishing variables and species. The eigenvalues of the compromise correspond to 74%, 18% and 7% of total variance, respectively (Fig 5). To summarize the interpretation of the compromise, the three axes can be mainly associated with each of the environmental parameters: axis 1 with SST, axis 2 with Chl a and axis 3 with fishing effort. To a lower extent, Chl a was mainly correlated with factor plane constituted by axes 2 and 3, and fishing effort (OpNu) with the factorial plane of axes 1–2 and 1–3 (Fig 5).

### Species assemblages

**Fig 6:**
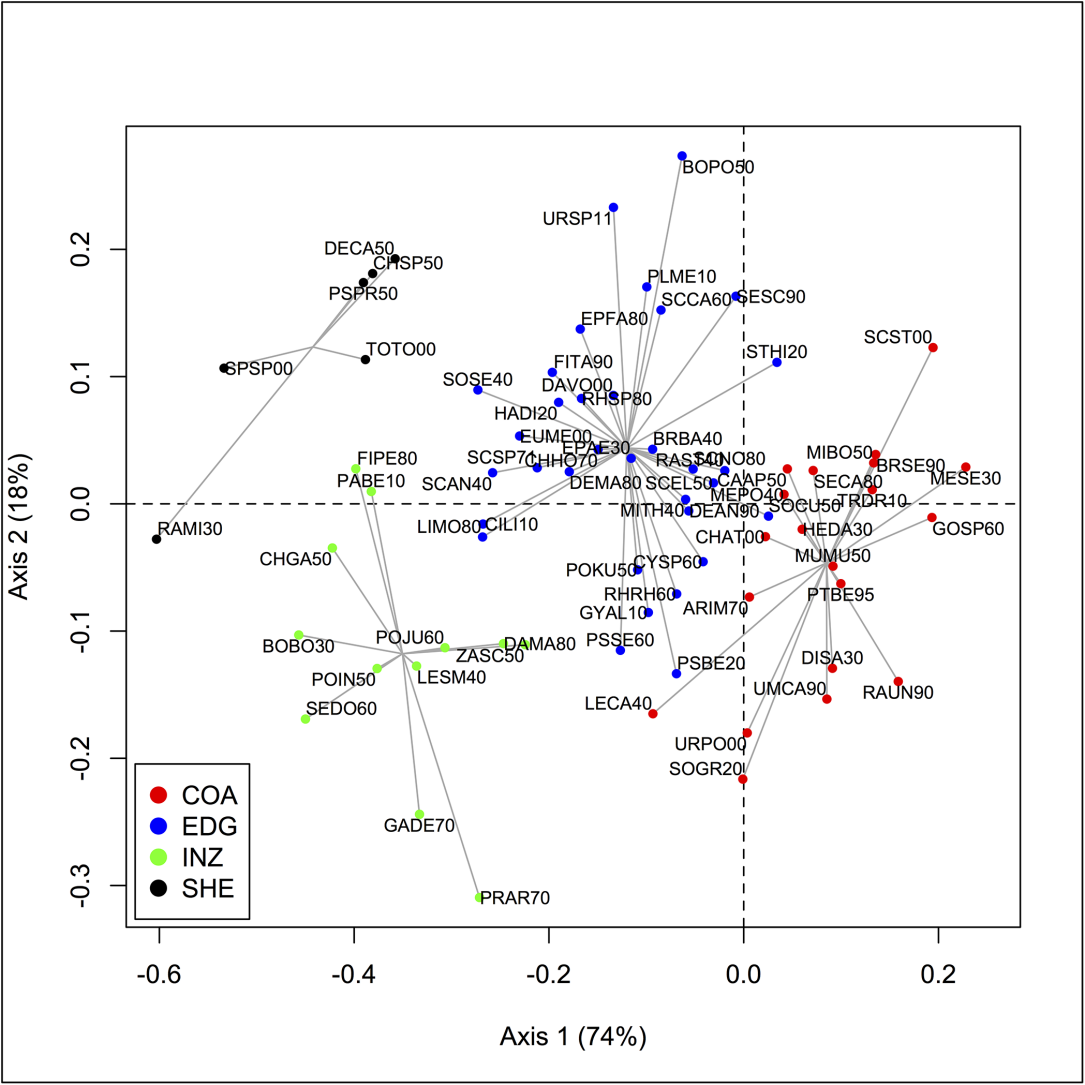
Projections of groundfish species variables and assemblage groups projected on the compromise space. The first factor plane (axes 1 and 2) with the main assemblage species obtained by hierarchical cluster analysis (UPGMA, see the methods section): coastal (COA), intermediate (INZ), shelf edge (EDG) and deeper assemblages (SHE).

Hierarchical clustering method on the species coordinates on the three first axes of the compromise analysis helps to identify four main assemblages (Fig 6 and S2 Text). They are composed of two coastal and soft muddy bottoms species assemblages located up to 50 m. The coastal assemblage (COA) is encountered in sandy bottoms at depths below 20 m. The intermediate assemblage (INZ), also found in soft, muddy, sandy and near rocky bottoms but encountered between 20–50 m depth (limits of the upper-shelf).

Two other assemblages encountered in deeper areas (> 50 m depth), consist of species living in muddy, sand-muddy and near rocky bottoms. The assemblage of the continental shelf edge (EDG) is encountered within 50–80 m limits. The last deeper assemblage (SHE) is encountered in the area of the continental shelf at depths exceeding 80 m.

First, coastal assemblages (*i*.*e*. COA and INZ) consist mainly of species found on soft bottoms sedimentary types (sandy, sand-muddy and muddy). Both osteichthyes and chondrichthyes families are represented. Osteichthyes are composed of Sparidae (*Pagellus bellottii*, *Diplodus sargus*), Sciaenidae (*Umbrina canariensis*), Soleidae (*Microchirus boscanion*) and some others species (*e*.*g*. *Scorpaena stephanica*, *Merluccius senegalensis*). Chondrichthyes species are composed by the smooth dogfish (*Mustelus mustelus*), Rhinobatidae (*Rhinobatos rhinoba*tos, *Zanobatus schoenleinii*) and some rays (e.g. *Dasyatis marmorata*, *Raja undulata*) (S2 Text). The main coastal assemblages encountered in near rocky bottoms are dominated by osteichthyes families of Sparidae, Scorpaenidae, Merlucciudae, Haemulidae, Soleidae, Chlorophthalmidae (Appendix S2). Some chondrichthyes are also encountered in these coastal assemblages, namely barbeled hound shark (*Leptocharias smithii*) and spotted skate (*Raja straeleni*).

Second, deep assemblages (EDG and SHE) found beyond 100 m consists of a group of osteichtyes families of Serranidae (*Epinephelus aeneus*, *Serranus scriba*), Sciaenidae (*Pseudotolithus senegalensis*), Soleidae (*Dicologoglossa cuneate*), Batrachoididae (*Halobatrachus didactylus*), Monacantidae (*Stephanolepis hispidus*) and a ray species (*Raja miraletus*) that were encountered on soft bottoms. However, the main species found near sedimentary rock type on these depths consist of a group of seven orders of Osteichthyes species (Perciformes, Pleuronectiformes, Tetraodontiformes, Scopaeniformes, Ophidiiformes, Dactylopteriformes and Sygnathiformes) and chondrichthyes by two orders (Carcharhiniformes and Torpediniformes) (S2 Text).

### Compromise on species assemblages variables

On the first factorial plane (Fig 6), the species assemblages are evenly distributed, with the coastal assemblage (COA) associated with positive values on the first axis (*i*.*e*. low SST, Fig 5). We note an opposition of the coastal assemblage (COA) to those deeper (SHE) in the negative part of this axis (i.e. high SST, Fig 5). On the second axis (mainly associated to Chl a, Fig 5), deeper species assemblages (EDG and SHE, higher Chl a, Fig 5) are opposed to coastal species assemblages (COA and INZ, lower Chl a, Fig 5), revealing a longitudinal gradient. On the third axis associated to the fishing pressure, the species distribution has no clear pattern according to these four assemblages (not shown).

### Trajectories of environment-fishing conditions and species assemblages

**Fig 7:**
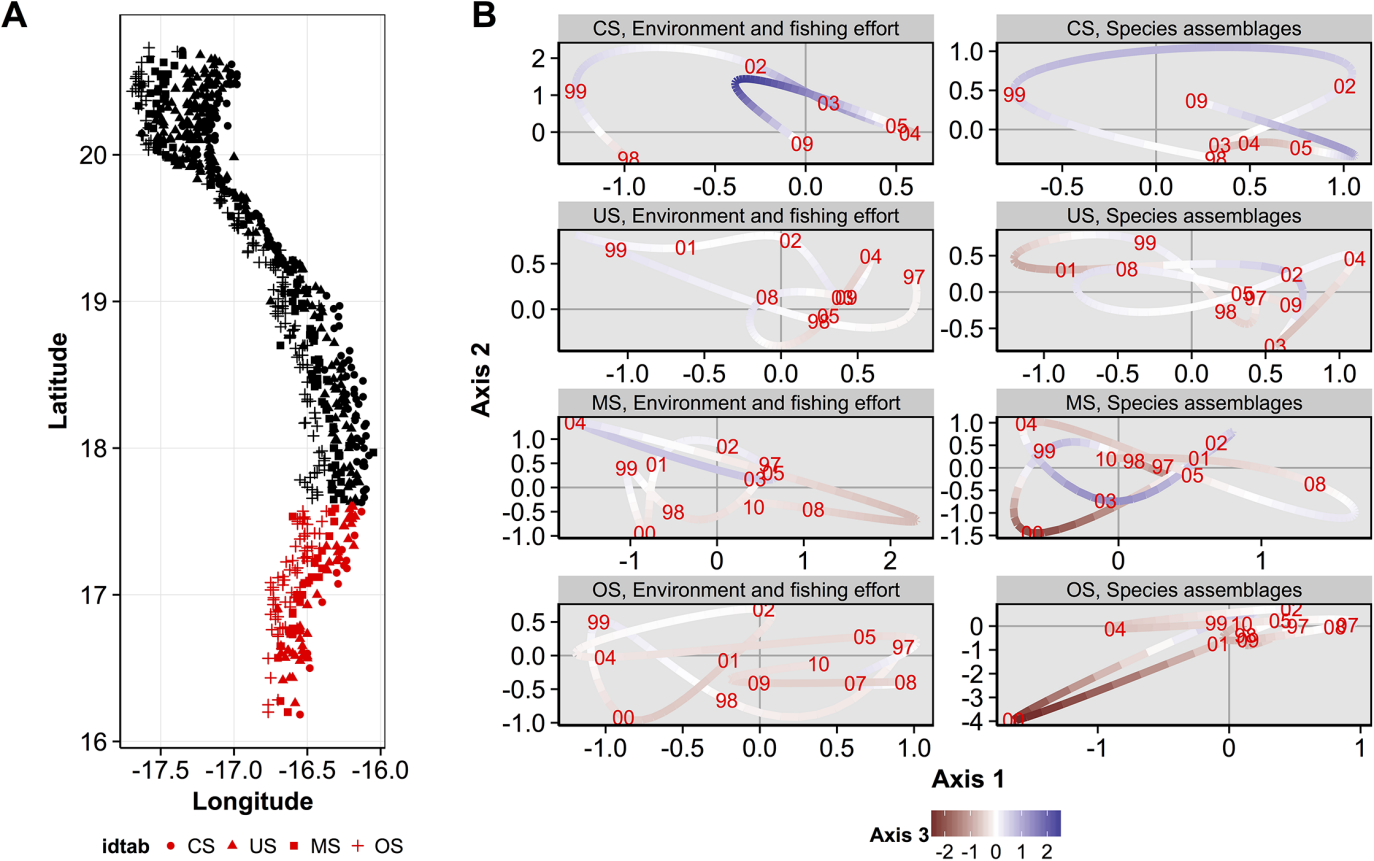
Trajectories coordinates of environmental-fishing and assemblages variables for sampling years on the three first axis of the compromise for each depth stratum in the southern area. (A) Locations of samples according to the depth stratum: coastal shelf (CS), upper shelf (US), mid-shelf (MS) and outer shelf (OS). (B) Trajectory plots of the environmental-fishing variables (left panel) and groundfish assemblages (right panel) are represented in the first plane (axes 1 and 2), with colors associated to the coordinates of axis 3 (see the bottom legend on the figure).

**Fig 8:**
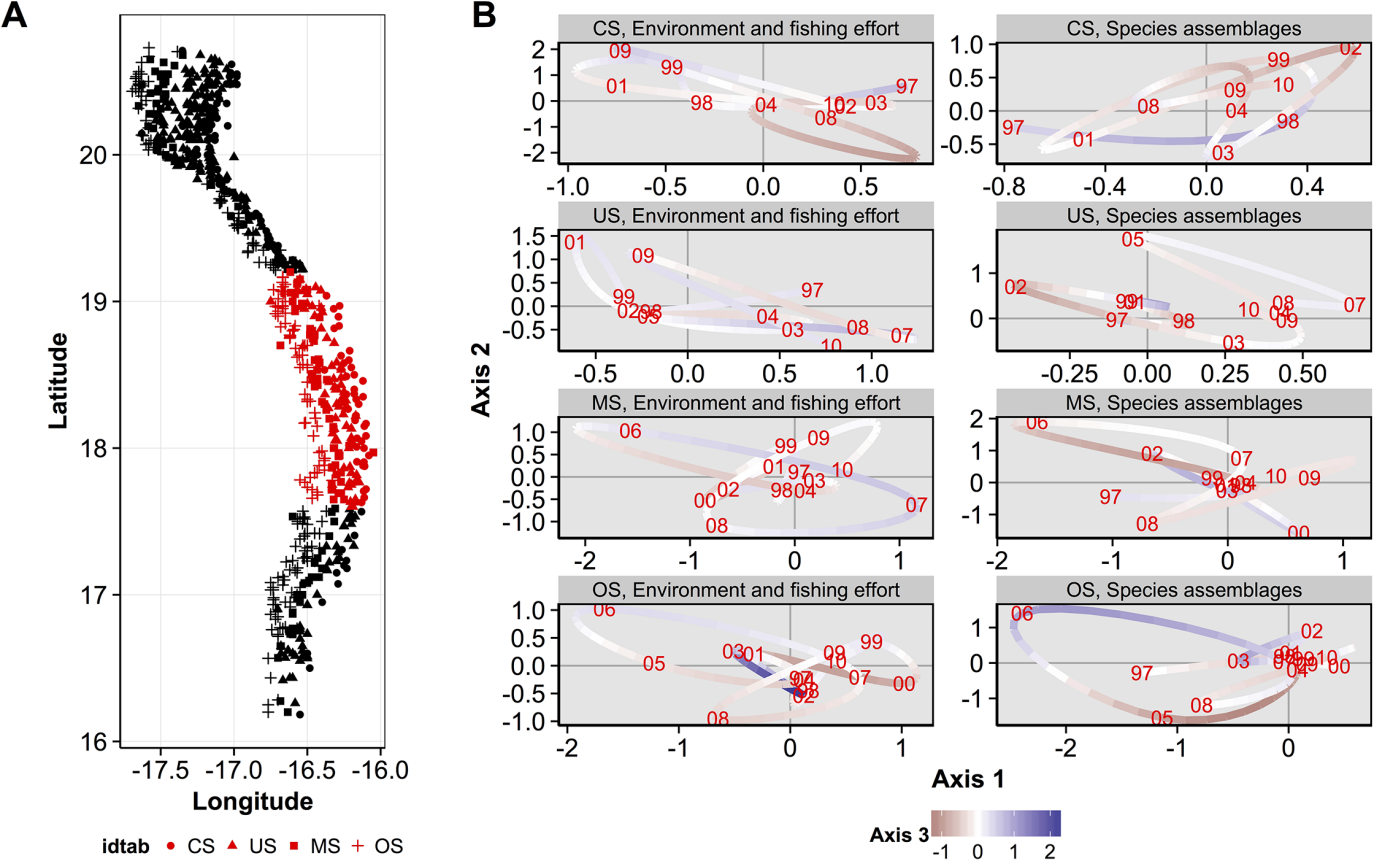
Trajectories coordinates of environmental-fishing and assemblages variables for sampling years on the three first axis of the compromise for each depth stratum in the central area. (A) Locations of samples according to the depth stratum: coastal shelf (CS), upper shelf (US), mid-shelf (MS) and outer shelf (OS). (B) Trajectory plots of the environmental-fishing variables (left panel) and groundfish assemblages (right panel) are represented in the first plane (axes 1 and 2), with colors associated to the coordinates of axis 3 (see the bottom legend on the figure).

**Fig 9:**
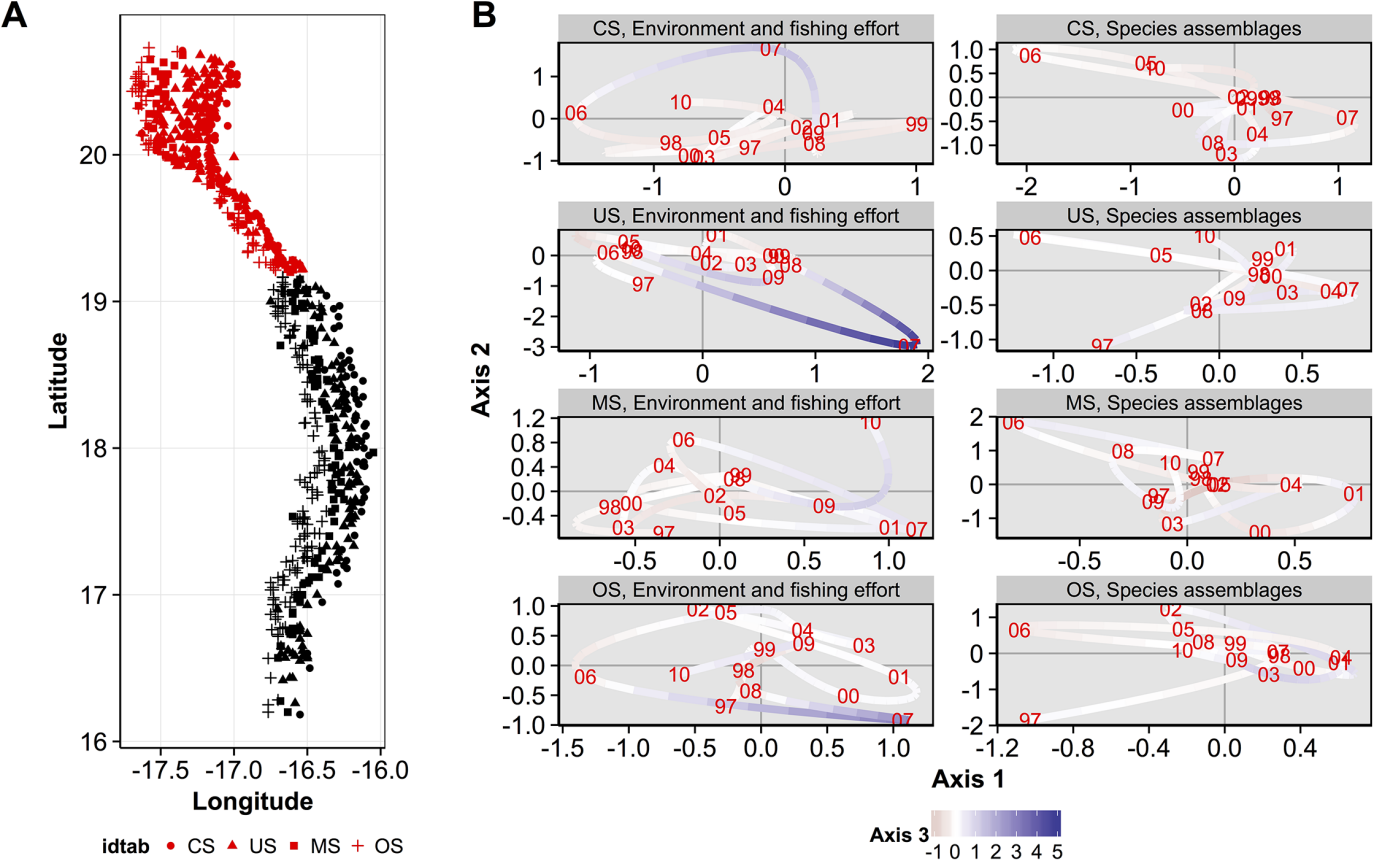
Trajectories coordinates of environmental-fishing and assemblages variables for sampling years on the three first axis of the compromise for each depth stratum in the northern area. (A) Locations of samples according to the depth stratum: coastal shelf (CS), upper shelf (US), mid-shelf (MS) and outer shelf (OS). (B) Trajectory plots of the environmental-fishing variables (left panel) and groundfish assemblages (right panel) are represented in the first plane (axes 1 and 2), with colors associated to the coordinates of axis 3 (see the bottom legend on the figure).

To provide the main trends of the spatio-temporal variability of environmental-fishing conditions and groundfish assemblages, their respective trajectories were projected on the three first axes of the compromise for each sampling years (Figs 7, 8 and 9), separately for the three main northern, central, southern geographical areas and for each bathymetric stratum (Figs 1 and 2). For these trajectories (Figs 7, 8 and 9), a year point results from the mean coordinates on each axis of stations belonging to a given geographical area and bathymetric stratum. For better clarity, coordinates on the axis 3 are represented by a colored gradient (*i*.*e*. negative values are in red and positive values in purple, Figs 7, 8 and 9; and are associated to a low and high fishing effort, respectively, see Fig 5). Contraction of the points cloud, its stretching and the proximity or remoteness along one or more axes gives information about the relationships between environmental-fishing (Fig 5) and assemblages variables for the years analyzed in a given geographical area and bathymetric stratum (Figs 7, 8 and 9). Notably, when the patterns of trajectories for assemblages are similar to the environment-fishing ones for some years, it can be considered that changes in assemblages are related to the environmental-fishing conditions. We remind that the axis 1 is associated to SST, axis 2 to Chl a and axis 3 to the fishing effort OpNu (Fig 5).

Overall for the three geographic areas, the trajectories between environment and fishing conditions and assemblages did not match for all the entire time-series analyzed, but interestingly for some particular years.

In the southern zone (Fig 7A), for the coastal CS stratum (Fig 7B), we can notice shifts in trajectories of the environmental variables between 1998 to 2005, with higher SST from 1998 (*i*.*e*. negative part of the axis 1, Figs 5 and 7B left panel) towards lower SST until 2005 (*i*.*e*. positive part of the axis 1, Figs 5 and 7B). This is coupled with an increase of Chl a during from 1999 to 2002 followed by a decrease in 2003 to 2005 (see trajectories on the positive part of the axis 2 which is associated to high Chl a, Figs 5 and 7B). In addition, fishing effort increased until 2003 before decreasing in 2004 and 2005 (see the colored gradient of axis 3 Fig 7B, where red is low and purple is high fishing effort according to the negative/positive values of coordinates on this axis, Fig 5). These complex changes in environmental-fishing conditions were associated to those of assemblages which trajectories were similar in 1999, 2003 to 2005 (Fig 7B, right panel).

In the central area (Fig 8A), the MS stratum (Fig 8B) also presented contrasted situations through time of environmental-fishing conditions between 2003 to 2008 associated with changes of assemblages. 2003 and 2004 were characterized by mean levels of SST and Chla (*i*.*e*. positioned at the center of the plane, Figs 5 and 8B), followed by an increase until 2006 (*i*.*e*. located on the upper left part of the plane, Fig 8B) and a decrease in fishing effort (*i*.*e*. coordinates from red to white colors–high to mean levels of fishing effort, Figs 5 and 8B). Then a shift of these three variables occurred in 2007 (located at the opposite of 2006 on the plane, Fig 8B) with a decrease in SST, Chl a and an increase in fishing effort (Figs 5 and 8B). Finally, again SST increased and fishing effort decreased in 2008 (Fig 8B), with Chl a remained low (*i*.*e*. 2008 is located at the lower left part of the plane, Fig 8B). Trajectories of assemblages were similar during 2003 to 2008, and thus linked to the above changes in environmental and fishing conditions, except in 2007 which is located at a mean position on the plane (Fig 8B) which suggests that assemblages were not strongly affected by the 2007’s shift.

In the northern area (Fig 9A), the US stratum in 2007 showed a different trajectory than the other years (Fig 9B), with low SST and Chl a (i.e. positive part of axis 1 and negative part of the axis 2, respectively, Figs 5 and 9B left panel), and high fishing effort (positive part of the axis 3, Figs 5 and 9B). With a similar trajectory, the assemblage in 2007 appeared to be linked in mean to these specific conditions (positive part of axis 1, Fig 9B right panel).

## Discussions

### Environment and groundfish assemblages

First, the simultaneous analysis of groundfish species assemblages’ abundance and the environment-fishing variables by the STATICO methods highlights in the interstructure analysis two groups of depth strata: coastal strata CS and US, and deeper strata MS and OS. Second, it helps to identify four major groundfish assemblages on the Mauritanian continental shelf on the compromise analysis. They correspond to Sciaenidae communities (COA) of the coastal zone (CS and US), and to Sparidae communities (INZ) in the upper-shelf (US), respectively. In the deeper parts of the continental shelf (MS and OS), EDG and SHE assemblages would correspond to the community near rocky outcrops, consisted of various families (Ophidiidae, Sparidae, Scorpaenidae, Sciaenidae, Merlucciidae and Centrophoridae). These assemblages are congruent with those described by several works in the MEEZ [46,58,59,60,61], and the effect of bathymetric gradient on species composition is consistent with those observed in areas of the world ocean [62,63,64,65,66,67,68]. Third, in some specific years and not for the entire time series analysed, the groundfish assemblages were strongly associated to hydrologic conditions predominating in the bathymetric zones (Figs 7, 8 and 9), characterized by permanent (notably in the Cap Blanc area [31]) or seasonal upwellings and contrasted SST and Chl a depending the geographical area (Figs 1 and 2). For instance, in the warm season, wind direction changes and warm surface waters comes from the south. These water masses less saline and poor in nutrients stem from the intensification of the Guinea current (SACW) towards Cap Blanc area [69,70,71,72], and might thus influence the food web until the groundfish level. Overall, the structure and distribution of groundfish assemblages of MEEZ follow a coast-offshore gradient and would depend on the local environmental conditions that vary along latitude (Figs 1 and 2) and between years (Figs 7, 8 and 9).

### Fishing effort and groundfish assemblages

Fishing effort was mainly higher in the northern area, but intermediate levels occurred in the central and southern areas (Fig 2C). The fact that the northern area is attractive for fishing may be due to hydrological conditions (permanent upwelling) promoting phytoplankton blooms (high primary production) and inducing a more productive system. While our results showed that the response of the spatial structuring of the groundfish assemblages was mainly driven by SST and Chl a in the studied area (Figs 5, 7, 8 and 9), they also highlighted the effect of fishing effort in some specific years and bathymetric strata, particularly in 2007 in the US strata of the northern area. Several studies taking into account the effects of fishing on the resources, conducted in the north-west Africa in general and in the MEEZ in particular, mainly focused on a few species of economic interest. These studies found an impact on the trophic level on exploited groundfish assemblages [41,73]. In other regions, targeted species were affected more directly by fishing pressure and changes in the structure of groundfish assemblages [74,75,76,77].

### Spatio-temporal variability of groundfish assemblages

Interestingly, for bathymetric stratum considered separately within each of the three geographic areas, the trajectories between environmental and fishing conditions and assemblages matched for some particular years and areas, and not for all the entire time-series analyzed in the MEEZ (Figs 7, 8 and 9). First when the trajectories matched, it revealed a complex effects of both SST, Chl a (associated to axes 1 and 2) and fishing effort (axis 3) on assemblages in some specific years. Such influence may be mainly due to years but also to seasonal variations in hydrological conditions, distribution of biological productivity in the MEEZ and in the northwest African region in general [35,78,79,80,81,82]. Notably, the presence of temperate and tropical species affinities attests to the seasonal variability of local water masses in the area constituting a biogeographic corridor, such as in other upwelling ecosystems [43,62,83,84].

Second when the trajectories did not match for some years, it could be linked to lags in response of assemblages faced to changes in conditions and/or to the fact that they can sustain despite these changes. It can also suggest that other factors than those we have investigated (*i*.*e*. SST, Chl a, fishing effort) may act on fish assemblages. Indeed, it is known that three main drivers influence species distributions at different spatial scales: (i) abiotic constraints, (ii) dispersal and (iii) biotic interactions (e.g. predation, competition and facilitation, see [85,86]). Ignoring in statistical analysis a combination of these explicative variables may lead to a certain part of unexplained variability [87,88]. However, some of these variable are not always quantified for every species in natural assemblages (e.g. biotic interactions or dispersal limitations), especially for groundfish species. When biotic information is not available, it is thus usual to only take into account abiotic variables in analyses. In our case, the three variables considered already explained an important part of the variability (the first axis of the interstructure explained 44% of the total variance, Fig 4; and the first three axes of the compromise represented 99% of the variance of the common structure on environmental-fishing and species variables, Fig 5).

## Conclusions

This study investigated from a quantitative point of view simultaneously the effects of both environmental and fishing factors on spatio-temporal dynamics of the species composition and abundance of exploited fish assemblages (both target and non-target species). We analyzed a huge data set of 854 hauls over 14 years by mean of the STATICO method in order to highlight key features of the studied system. In the Mauritanian Exclusive Economic Zone, abiotic factors investigated (*i*.*e*. SST, Chl a and fishing effort) drove the spatial structure of four main demersal assemblages differently in some specific years according to the area (latitude and depth strata). In further studies on exploited resources, it would be necessary to investigate other groups of fish species (*i*.*e*. pelagic), and taxonomic groups (i.*e*. cephalopods) due to their socio-economic importance in many fisheries. Indeed, several kinds of fisheries (artisanal and industrial fisheries on demersal and pelagic resources) constitute a pole of activities in Mauritania [32,33] and other areas worldwide. These analyses and results may provide to managers, scientist and fishers an important approach to assess the spatial-temporal dynamics of exploited assemblages under different degree of stability or shifting conditions of fishing and environment.

## Supporting Information

S1 File R script and data files. The zip file contains i) the R script used to analyze the stability and the variability of groundfish species assemblages abundance, environmental and fishing parameters in the Mauritanian Exclusive Economic Zone, ii) three data files (.txt format) of the list of groundfish species names, family names, their code, assemblage to which they belong, sedimentary and bottom types (spec.txt); environmental and fishing variables (env.txt); groundfish species abundance (bio.txt).
(ZIP)

S1 Text Description of the STATICO method following Thioulouse et al. (2004). (DOCX)

S2 Text Fish assemblages, with species list, identified by mean of average linkage classification (UPGMA, see methods section), with their sedimentary types and depth stratum. (DOCX)
