# Supplementary material for: Spatio-Temporal Dynamics of Exploited Groundfish Species Assemblages Faced to Environmental and Fishing Forcings: Insights from the Mauritanian Exclusive Economic Zone
Source: PLoS One. 2015 Oct 27;10(10):e0141566. doi: 10.1371/journal.pone.0141566 (PMC4623501; doi:10.1371/journal.pone.0141566)
Supplement: S1 Text — (DOCX) [file pone.0141566.s002.docx]

**S1 Text: Description of the STATICO method following Thioulouse et al. (2004).**

Consider a list of k pairs $\left\{ \left( X_{i},Y_{i} \right):1\leq i\leq k \right\}$ of tables. In our case, each pair is associated with a depth stratum. For one depth stratum *i*, *X_i_* (*n* × *p*) is a table of *p* environmental or fishing effort variables sampled *n* times, and *Y_i_* (*n* × *q*) corresponds to *q* species abundance sampled *the same way* (*n* is the year number in our case). *D_p_* (*p* × *p*) and *D_q_* (*q* × *q*) are the diagonal matrices of column weights (variables) and *D_n_* (*n* × *n*) is the diagonal matrix of row weights (years). STATICO results from merging two multivariate analysis methods: Co-inertia analysis, and Partial Triadic Analysis.

(1) Co-inertia analysis of a pair of tables:

The co-inertia analysis of *X_i_* and *Y_i_* is the eigen analysis of the matrix *Z_i_*:

*Z_i_* = *X_i_^T^D_n_Y_i_D_q_Y_i_^T^D_n_X_i_D_p_*

Following Dray et al. (2003), the analysis of *Z_i_* gives the common geometry of the two tables. It searches for axes that maximize the covariance between the coordinates of the rows (sampling years) of the two tables (environmental and fishing effort variables, and species abundance).

(2) Partial Triadic Analysis (PTA) of a series of tables:

Consider a series of tables $\left\{ Z_{i}:1\leq i\leq k \right\}$ with *p* rows and *q* columns (identical); *D_p_*(*p* × *p*) and *D_q_*(*q* × *q)* are the diagonal matrices of row and column weights, respectively. The analysis of this series of tables by PTA consists in three steps.

(2-a) Interstructure analysis:

A matrix of scalar products is computed between all the tables *Z_i_*. It is the vector covariance matrix (Escoufier, 1973), of general term

*Cov*(*Z_i_* ; *Z_j_* ) = *Trace*(*Z_i_^T^D_n_Z_j_D_p_*) for *i*=*1*,...,*k* and *j*=*1*,...,k.

The vector variance of table *Z_i_* is:

*Vav(Z_i_)* = *Trace*(*Z_i_^T^D_n_Z_i_D_p_*) for *i*=*1*,...,*k*

And the matrix of vector correlations is defined by:

*Rv*(*Z_i_* ; *Z_j_* ) = *Covv*(*Z_i_* ; *Z_j_* ) × [*Vav*(*Z_i_*) × *Vav*(*Z_j_*)]^-0.5^ for *i*=*1*,...,*k* and *j*=*1*,...,*k*

The eigen analysis of this matrix gives a first eigenvector α, whose components are used as weights to compute the compromise table:

$$Z_{c}=\sum_{k} \alpha_{k}Z_{k}$$

(2-b) Compromise analysis:

The principal component analysis (PCA) of $Z_{c}$ gives factor maps that are used to interpret the similarities or dissimilarities between original tables, in reference with common structures revealed by the PCA.

(2-c) trajectories:

The rows (*i*.*e*., years) and columns (*i*.*e*., variables) of the initial tables are then projected onto the factor maps.

STATICO consists in carrying PTA on the series of co-inertia matrices associated with the original pairs $\left\{ \left( X_{i},Y_{i} \right):1\leq i\leq k \right\}$ of tables. In our case, species, environmental and fishing effort variables and sampling years can be projected (trajectories) as additional elements on the compromise principal axes in order to summarize the reproducibility of the structure across the depth strata.

STATICO is quite simple to run because it requires only matrix and eigensystem computation. However, the variable scaling and the matrix of row and column weights have to be chosen with great care because they imply different ecological considerations (Dray et al. 2003). In our case, the separate analysis of each table was performed using a PCA because the species responses to the environmental and fishing effort variables are roughly linear (this was previously carefully verified graphically using a scatterplot). Then the PCA for the environmental and fishing effort variables was normalized because of the large scale variability. The PCA of the species abundances was performed on the log +1 transformed data to reduce the data variance as well as to simplify the graphical representation. Weighting matrices were considered uniform because the sampling method was homogeneous throughout the time and the taxonomic identification was performed carefully.

References

Dray, S., D. Chessel, and J. Thioulouse. 2003. Co-inertia analysis and the linking of ecological data tables. Ecology 84(11), 3078–3089.

Escoufier, Y. 1973. “ Le traitement des variables vectorielles”. Biometrics (International Biometric Society) 29(4) :751-760.

Thioulouse, J., M. Simier, and D. Chessel. 2004. Simultaneous analysis of a sequence of paired ecological tables. Ecology 85(1): 272–283.
