# Supplementary material for: Spatio-Temporal Dynamics of Exploited Groundfish Species Assemblages Faced to Environmental and Fishing Forcings: Insights from the Mauritanian Exclusive Economic Zone
Source: PLoS One. 2015 Oct 27;10(10):e0141566. doi: 10.1371/journal.pone.0141566 (PMC4623501; doi:10.1371/journal.pone.0141566)
Supplement: S2 Text — (DOCX) [file pone.0141566.s003.docx]

**S2 Text**: **Fish assemblages, with species list, identified by mean of average linkage classification (UPGMA), with their sedimentary types and depth stratum (CS: Coastal, US: Upper shelf, MS: Mid-shelf and OS: Outer shelf).**

| Depth strata | Sedimentary types | Taxonomic names | Family |
| --- | --- | --- | --- |
| CS | Muddy | *Merluccius senegalensis* | Merlucciudae |
|  |  | *Pseudotolithus senegalensis* | Sciaenidae |
|  | Rocky | *Capros aper* | Caproidae |
|  |  | *Merluccius polli* | Merlucciudae |
|  |  | *Scorpaena elongata* | Scorpaenidae |
|  |  | *Helicolenus dactylopterus* |  |
|  |  | *Pontinus kuhlii* |  |
|  |  | *Lepidotrigla cadmani* | Triglidae |
|  | Sandy | *Pterothrissus belloci* | Albulidae |
|  |  | *Arnoglossus imperialis* | Bothidae |
|  |  | *Chlorophthalmus atlanticus* | Chlorophthalmidae |
|  |  | *Gobiidae* | Gobiidae |
|  |  | *Gymnura altavela* | Gymnuridae |
|  |  | *Branchiostegus semifasciatus* | Malacanthidae |
|  |  | *Psettodes belcheri* | Psettodidae |
|  |  | *Raja straeleni* | Rajidae |
|  |  | *Raja undulata* |  |
|  |  | *Rhinobatos rhinobatos* | Rhinobatidae |
|  |  | *Scorpaena normani* | Scorpaenidae |
|  |  | *Scorpaena stephanica* |  |
|  |  | *Serranus cabrilla* | Serranidae |
| *Microchirus theophila* | Soleidae |  |  |
| US | Sandy | *Dentex angolensis* | Sparidae |
|  |  | *Diplodus sargus* |  |
|  |  | *Mustelus mustelus* | Triakidae |
|  |  | *Uranoscopus polli* | Uranoscopidae |
|  | Sand-muddy | *Cynoglossus* spp. | Cynoglossidae |
|  |  | *Grammoplites gruveli* | Platycephalidae |
|  |  | *Umbrina canariensis* | Sciaenidae |
|  |  | *Microchirus boscanion* | Soleidae |
|  |  | *Dicologoglossa cuneata* |  |
|  |  | *Trachinus draco* | Trachinidae |
|  | Muddy | *Priacanthus arenatus* | Priacanthidae |
|  |  | *Zanobatus schoenleinii* | Rhinobatidae |
|  | Rocky | *Selene dorsalis* | Carangidae |
|  |  | *Fistularia petimba* | Fistulariidae |

To be continued

| **Depth strata** | **Sedimentary types** | **Taxonomic names** | **Family** |
| --- | --- | --- | --- |
| US | Sandy | *Dasyatis marmorata* | Dasyatidae |
|  |  | *Pomadasys incisus* | Haemulidae |
|  |  | *Pomadasys jubelini* |  |
|  |  | *Leptocharias smithii* | Leptochariidae |
|  |  | *Boops boops* | Sparidae |
|  |  | *Pagellus bellottii* |  |
|  |  | *Chelidonichthys gabonensis* | Triglidae |
|  | Sand-muddy | *Galeoides decadactylus* | Polynemidae |
| MS | Sandy | *Halobatrachus didactylus* | Batrachoididae |
|  |  | *Bothus podas* | Bothidae |
|  |  | *Chaetodon hoefleri* | Chaetodontidae |
|  |  | *Citharus linguatula*  *Dactylopterus volitans* | Citharidae  Dactylopteridae |
|  |  | *Fistularia tabacaria* | Fistulariidae |
|  |  | *Eucinostomus melanopterus* | Gereidae |
|  |  | *Plectorhinchus mediterraneus* | Haemulidae |
|  |  | *Stephanolepis hispidus* | Monacanthidae |
|  |  | *Brotula barbata* | Ophidiidae |
|  |  | *Rhinobatos* spp. | Rhinobatidae |
|  |  | *Scorpaena angolensis* | Scorpaenidae |
|  |  | *Scorpaena* spp. |  |
|  |  | *Scyliorhinus canicula* | Scyliorhinidae |
|  |  | *Epinephelus alexandrinus* | Serranidae |
|  |  | *Epinephelus aeneus* |  |
|  |  | *Serranus scriba* |  |
|  |  | *Solea senegalensis* | Soleidae |
|  |  | *Lithognathus mormyrus* | Sparidae |
|  |  | *Uranoscopus* spp. | Uranoscopidae |
|  | Sand-muddy | *Dentex maroccanus* | Sparidae |
| OS | Sandy | *Pseudupeneus prayensis* | Mullidae |
|  |  | *Raja miraletus* | Rajidae |
|  |  | *Dentex canariensis* | Sparidae |
|  | Sand-muddy | *Chilomycterus orbicularis* | Diodontidae |
|  |  | *Sphoeroides spengleri* | Tetraodontidae |
|  |  | *Torpedo torpedo* | Torpedinidae |
